# Supplementary material for: Evolution of the Standard Genetic Code
Source: J Mol Evol. 2021 Jan 24;89(1):19–44. doi: 10.1007/s00239-020-09983-9 (PMC7884377; doi:10.1007/s00239-020-09983-9)
Supplement: Supplementary file 1 — Supplementary file1 - Lexicon (PDF 87 KB) [file 239_2020_9983_MOESM1_ESM.pdf]

Expressions used with a special meaning:

0 +/- 1 PR – Paralogous mutational capture of a new codon, with the new assignment limited to amino acids separated by 0 or 1 places in the polar requirement series.

0 +/- 2 PR – Paralogous mutational capture of a new codon, with the new assignment limited to amino acids separated by 0, 1 or 2 places in the polar requirement series.

0 +/- 3 PR - Paralogous mutational capture of a new codon, with the new assignment limited to amino acids separated by 0, 1, 2 or 3 places in the polar requirement series.

0 +/- 4 PR - Paralogous mutational capture of a new codon, with the new assignment limited to amino acids separated by 0, 1, 2, 3 or 4 places in the polar requirement series.

Bayesian convergence – rapidly increasing probability for a hypothesis when multiple, particularly independent, observations are explained.

Chance utility – temporary or permanent change in a system without genetic inheritance; that is, a purely chemical system.

Coevo – coevolutionary mutational capture, with newly assigned amino acids having a biosynthetic relation to an existing one.

Coevo_PR – Coevo, but with new assignments constrained to favor a similar polar requirement.

Completion complexity – increased time and pathway complexity required for assignment of the last of 22 encoded functions.

Continuous wobble – wobble occurs continuously, throughout coding table history.

Decay – loss of a previously assigned triplet function.

Distance – progress value, varies from 0.0 to 1.0 as mean mutational distance between system triplets and identically assigned SGC triplets goes from random to the value for the SGC itself.

Distribution fitness - evolutionary success via varied individual fitness, the best of which are highly fit.

dPR – progress value varying from 0.0 to 1.0 as mean polar requirement difference between neighborhood triplets goes from random encoding to the (usually) smaller SGC value.

Initiation – assignment of 1 of 22 possible encoded functions to an unassigned triplet; abbreviated init.

Late wobble – evolutionary pathway in which assignments are to unique triplets, without wobble, then quick and complete adoption of a late-developing wobble mechanism.

Mutational capture – assignment of codon function related to that of an preexisting assigned neighborhood triplet, occurs only to unassigned triplets; abbreviated mut capt.

Mutational neighborhood – triplets related to a current one by single mutation, 9 neighbors per triplet.

Paralogous, paralogy, paralogical – mutational captures at random or by adaptation/selection, yielding amino acids with similar polar requirements assigned to related triplet codons.

Passage – one computational pass through a current coding table: passages are $\propto$ time.

Pdecay – probability that a chosen assigned triplet loses its assignment, per passage.

Pinit – probability of initial assignment for a random unassigned triplet, per passage.

Pmut – probability of mutational capture of an unassigned neighboring codon by an existing assignment, per passage.

Prand – probability that a new triplet assignment is random, rather than chosen from the SGC.

Pwob – probability of wobble if a codon triplet can be read either uniquely or via wobble, as for XYU.

Spacing – progress value varying from 0.0 to 1.0 as mean mutational distance between triplets with identical assignments goes from random to (usually) smaller SGC value.

Starting bloc selection – evolutionary success based on the fittest among a population being those just beginning the selected activity.
